# Supplementary material for: Differences in Immunogenicity of Three Different Homo- and Heterologous Vaccination Regimens against SARS-CoV-2
Source: Vaccines (Basel). 2022 Apr 20;10(5):649. doi: 10.3390/vaccines10050649 (PMC9145236; doi:10.3390/vaccines10050649)
Supplement: Supplementary file 1 [file vaccines-10-00649-s001.zip › supplement_SARS-CoV-2_vaccination_markewitz.pdf]

Supplementary Appendix to:

“Differences in immunogenicity of three different homo- and heterologous vaccination regimens against SARS-CoV-2”

Table of contents:

Methods: 2

Study population: 2

Analysis of data from participants 60 years of age and older: 2

Analysis of borderline or non-reactive results 2

Supplementary Figure legends: 3

Methods:

Study population:

As mentioned in the main body, there were three possible vaccination regimens for vaccinees who had received a first dose of ChAdOx: receiving two doses of ChAdOx (ChAdOx/ChAdOx a first dose of ChAdOx and a second one of Mod (ChAdOx/Mod), or a first dose of ChAdOx and a second one of BNT (ChAdOx/BNT). All vaccinees who had received a first dose of ChAdOx were offered a second dose of an mRNA-based vaccine (Mod or BNT). If this option was chosen, the vaccinees were pseudo-randomly assigned to receive either Mod or BNT, based on the availability of either vaccine on the date of the second vaccination. Recipients could not choose between Mod or BNT. The vaccinees also had the option to make an informed decision to receive a second dose of ChAdOx (even though this was not recommended by German health authorities and the use of ChAdOx is generally not recommended any more for recipients younger than 60 years in Germany). Due to the mentioned official recommendations by the Germany health authorities, there was an a priori bias towards older participants being more likely to receive ChAdOx/ChAdOx than any of the other two regimens.

For recruitment purposes, vaccinees were made aware of the possibility of a participation in the current study. Those willing to participate were asked to provide basic information (age, sex, potential immunosuppression (if known)). This information was used to invite vaccinees to participate. The goal was to achieve three cohorts made up of recipients of the three aforementioned vaccination regimens, each cohort consisting of at least 100 recipients of equal distribution of sex and age. No formalized considerations of power and sample size were undertaken beforehand (rendering the selected cohorts a sample of convenience). Rather, as many participants as possible were recruited that still fit abovementioned distribution.

Analysis of data from participants 60 years of age and older:

Among the 36 participants 60 years of age and older (mean: 63.1±2.8; range: 60-70 years old), 21 (58.3 %) were female. Of the 36, 26 (72.2 %) received ChAdOx/ChAdOx, 8 (22.2 %) received ChAdOx/Mod, and 2 (5.6 %) received ChAdOx/BNT (one of whom donated only the first blood sample).

In the age group 60 years and older, there were no differences between the sexes in any of the examined markers, neither after the first, nor the second dose of the vaccination. The comparison of the different vaccination regimens in this age group yields no significant differences after the first dose of the vaccination and the same trend for ChAdOx/ChAdOx to result in markedly lower levels of all examined markers after the second vaccination (except for IFN-γ via IGRA, for which there is only a statistical trend), compared to the mRNA-based regimens. Due to the small sample size, this difference is statistically significant mainly for the comparisons between ChAdOx/ChAdOx and ChAdOx/Mod (anti-S1 IgG: $p = 0.0003$; IgA: $p = 0.001$; neutralizing antibodies: $p = 0.02$, IFN-γ via IGRA: $p = 0.068$; see Figure S1).

Due to the small sample size of participants 60 years and older, these comparisons might have been underpowered and therefore possible effects of old age underestimated.

Analysis of borderline or non-reactive results

For all examined markers, we saw results that were below the cutoff for reactivity of the individual test 14 days after the second dose of the vaccination: One participant (having received ChAdOx/ChAdOx) tested borderline for anti-S1 IgG. Eighteen participants tested

borderline for anti-S1 IgA (of which all but one, who had received ChAdOx/BNT, had received ChAdOx/ChAdOx) and 31 tested non-reactive (of which two had received ChAdOx/BNT, one had received ChAdOx/Mod, and 28 had received ChAdOx/ChAdOx). One of the anti-S1 IgA negative participants had reported receiving Ciclosporin and Dupilumab, and another (who also tested borderline for neutralizing antibodies) received Rituximab. Among the population of participants 60 years and older, one participant, who tested negative in for both anti-S1 IgA and neutralizing antibodies, reported receiving Methotrexate. Four participants tested borderline in the surrogate neutralization assay (three having had received ChAdOx/ChAdOx, and one ChAdOx/BNT) and seven tested negative (all of which had received ChAdOx/ChAdOx). Four participants showed non-reactive levels of Interferon- γ in the IGRA (three with borderline results and one with a negative result), all of whom had received ChAdOx/ChAdOx.

Mann-Whitney *U* tests showed that those who had borderline or non-reactive results for a certain marker 14 days after the second dose, already had significantly lower results for the same marker after the first dose than those participants who had reactive results 14 days after the second dose. The differences are: anti-S1 IgA: 0.37±0.19 OD ratio vs. 0.62±0.39 OD ratio ($p<0.0001$); neutralizing antibodies: 4±4.4 % vs. 16±13.3 % ($p<0.0001$); and Interferon-γ via IGRA: 72±34.2 mIU/ml vs. 258±255.8 mIU/ml ($p = 0.018$). For anti-S1 IgG, this comparison is not possible due to the small sample size of borderline results after the second dose (n=1). Another Mann-Whitney *U* test revealed that there was no significant difference in age between participants with a borderline or non-reactive result for any marker 14 days after the second vaccination and those with reactive results for every marker ($p = 0.42$).

Supplementary Figure legends:

Figure S1: Comparison of the examined immune responses, both 12 weeks after the first vaccination (panels A-D) and 14 days after the second vaccination (panels E-H) in between the three vaccination regimes of the current study. Each plot represents a different marker: anti-S1 IgG (panels A and E), anti-S1 IgA (panels B and F), the inhibition by neutralizing antibodies (panels C and G), and T-cell response as measured via IGRA (panels D and H). The dotted horizontal lines represent the cutoff for reactivity of the different assays. Levels of significance: *** = p<0.001; ** = p<0.01; ns = not statistically significant

Supplementary Table legends:

Table S1: numerical makeup of the cohort, whose data went into statistical analysis. The percentages in the brackets represent the share of the stated number in relation to the total number of participants of the group defined by each column.
