# Supplementary material for: Differences in Immunogenicity of Three Different Homo- and Heterologous Vaccination Regimens against SARS-CoV-2
Source: Vaccines (Basel). 2022 Apr 20;10(5):649. doi: 10.3390/vaccines10050649 (PMC9145236; doi:10.3390/vaccines10050649)
Supplement: Supplementary file 1 [file vaccines-10-00649-s001.zip › tableS1.pdf]

	whole cohort	female	male	ChAdOx/ChAdOx	ChAdOx/Mod	ChAdOx/BNT
whole cohort	324	178	146	103	116	105
female	178 (54.9 %)			54 (52.4 %)	64 (55.2 %)	60 (57.1 %)
male	146 (45.1 %)			49 (46.6 %)	52 (44.8 %)	45 (42.9 %)
ChAdOx/ChAdOx	103 (31.8 %)	54 (30.3 %)	49 (33.6 %)			
ChAdOx/Mod	116 (35.8 %)	64 (36.0 %)	52 (35.6 %)			
ChAdOx/BNT	105 (32.4 %)	60 (33.7 %)	45 (30.8 %)			
Anti-NCP IgG pos.	9 (2.8 %)	4 (2.2 %)	5 (3.4 %)	2 (1.9 %)	4 (3.4 %)	3 (2.9 %)

Table S1: numerical makeup of the cohort, whose data went into statistical analysis. The percentages in the brackets represent the share of the stated number in relation to the total number of participants of the group defined by each column.
