# Supplementary figures and images for: Differences in Immunogenicity of Three Different Homo- and Heterologous Vaccination Regimens against SARS-CoV-2
Source: Vaccines (Basel). 2022 Apr 20;10(5):649. doi: 10.3390/vaccines10050649 (PMC9145236; doi:10.3390/vaccines10050649)

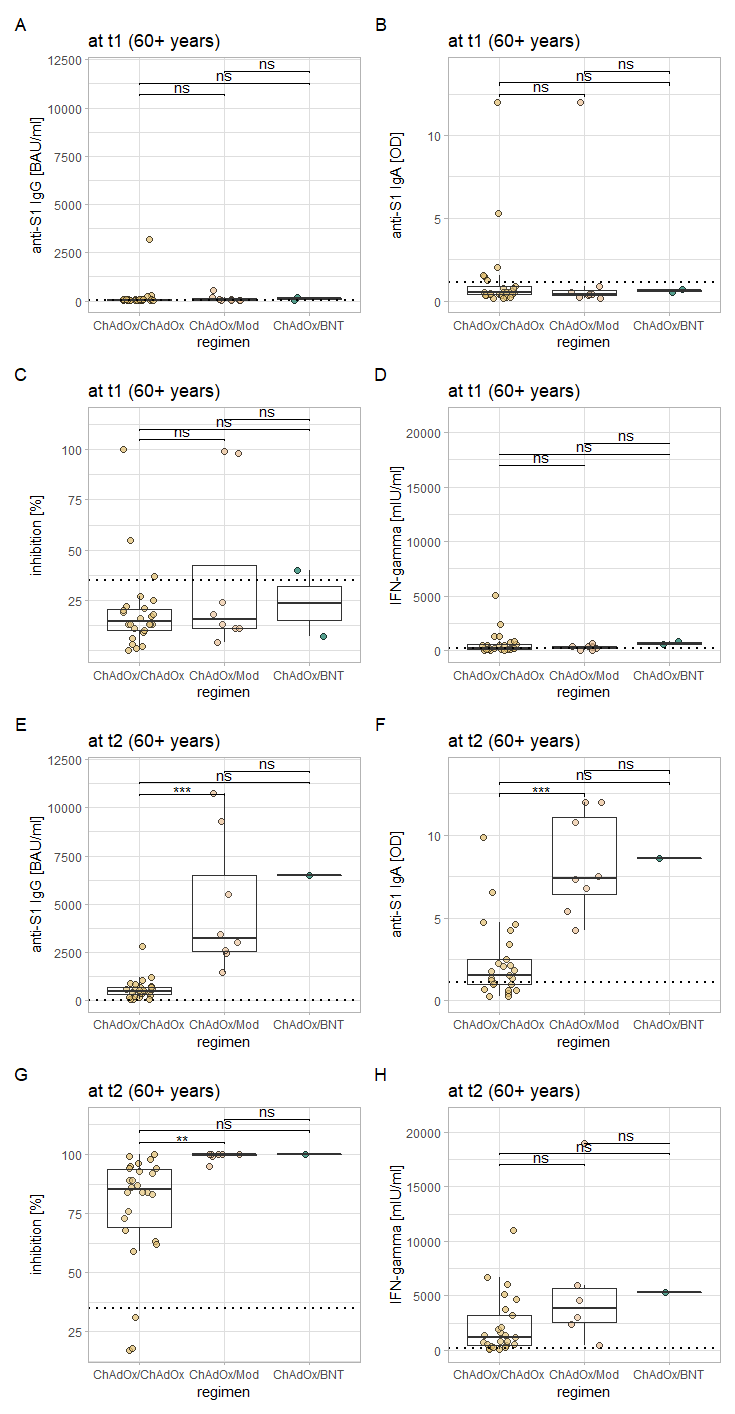

Supplement: Supplementary file 1 [file vaccines-10-00649-s001.zip › Figure_S1.tiff]
